# Moon phases and moon signs do not influence morbidity, mortality and long-term survival, after living donor kidney transplantation

**DOI:** 10.1186/s12906-017-1944-4

**Published:** 2017-09-04

**Authors:** A. Kleespies, M. Mikhailov, P. N. Khalil, S. Pratschke, A. Khandoga, M. Stangl, W. D. Illner, M. K. Angele, K. W. Jauch, M. Guba, J. Werner, M. Rentsch

**Affiliations:** Department of General-, Visceral-, Vascular- and Transplant Surgery, University of Munich, Marchioninistrasse 15, 81377 Munich, Germany

**Keywords:** Moon phases, Moon sign, Living donor kidney transplantation, Survival, Perioperative morbidity

## Abstract

**Background:**

Approximately 11% of the German population are convinced that certain moon phases and moon signs may impact their health and the onset and clinical course of diseases. Before elective surgery, a considerable number of patients look to optimize the timing of the procedure based on the lunar cycle. Especially patients awaiting living donor kidney transplantation (LDKT) commonly look for an adjustment of the date of transplantation according to the moon calendar. This study therefore investigated the perioperative and long-term outcome of LDKT dependent on moon phases and zodiac signs.

**Methods:**

Patient data were prospectively collected in a continuously updated kidney transplant database. Two hundred and seventy-eight consecutive patients who underwent LDKT between 1994 and December 2009 were selected for the study and retrospectively assigned to the four moon phases (*new-moon, waxing-moon, full-moon, and waning-moon*) and the corresponding zodiac sign (moon sign Libra), based on the date of transplantation. Preexisting comorbidities, perioperative mortality, surgical outcome, and long-term survival data were analyzed.

**Results:**

Of all LDKT procedures, 11.9, 39.9, 11.5, and 36.5% were performed during the new, waxing, full, and waning moon, respectively, and 6.2% during the moon sign Libra, which is believed to interfere with renal surgery. Survival rates at 1, 5, and 10 years after transplantation were 98.9, 92, and 88.7% (patient survival) and 97.4, 91.6, and 80.6% (graft survival) without any differences between all groups of lunar phases and moon signs. Overall perioperative complications and early graft loss occurred in 21.2 and 1.4%, without statistical difference (*p* > 0.05) between groups.

**Conclusion:**

Moon phases and the moon sign Libra had no impact on early and long-term outcome measures following LDKT in our study. Thus, concerns of patients awaiting LDKT regarding the ideal time of surgery can be allayed, and surgery may be scheduled independently of the lunar phases.

## Background

**Fig. 1:**
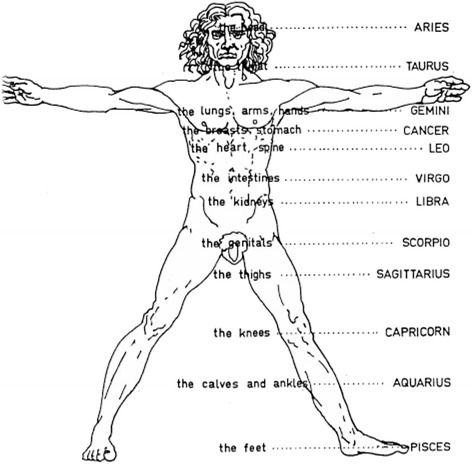
Anatomical-astrological human (drawing by © Alla Mikhailova). According to medical astrology, each organ (or organ system) is associated with a certain moon sign. Note, that the kidneys are assigned to the sign of *Libra*

In comparison to deceased donor transplantation, living donor kidney transplantation (LDKT) provides better patient and allograft survival. Reasons for this are shorter cold ischemic times, reduced time on the waiting list, and the opportunity for patient conditioning as well as to electively schedule surgery [1, 2]. Moreover, the decision to donate and to accept an organ can improve the well-being of both donor and recipient [3–6]. Nevertheless, the LDKT procedure itself can cause considerable emotional stress for the donor and the recipient. Since the German transplantation law does not permit altruistic organ donation, both donor and recipient frequently have a strong emotional connection (friends or relatives). Therefore, a positive outcome of the LDKT is generally expected and complications are considered unacceptable. However, between 6 and 25% of the complications following LDKT may be due to anatomical conditions (in particular vascular anatomy), AB0 incompatibility, and the recipient’s nutritional status [7]. As a consequence, it is not uncommon that donors and recipients wish to reduce the risk of an adverse outcome by selecting the best date for the transplantation procedure [4–6, 8–11]. In particular, patients and their relatives sometimes set the date by following superstition-based predictions. This includes the popular superstition that moon phases (new-, waxing-, full and waning moon) and moon signs (zodiac signs of the moon, which are believed to be associated with a certain part of the human body) might impact outcome after surgery. According to medical astrology, surgical interventions performed during full moon and moon signs of the corresponding surgical region might have a less favorable outcome (Fig. 1, [12]). So far, the timing of living donor kidney transplantation according to the lunar cycle and moon signs has not been investigated. The present work is the first to provide a structured analysis of the clinical impact of timing of an LDKT procedure according to moon phases and the moon sign Libra. The Results of this study might help surgeons to relieve patients’ concerns about the wrong timing of surgery.

## Methods

### Patients

Between 1994 and 2009, a total of 3722 kidney transplantations were performed at our transplant center. Among those, 278 consecutive pairs of adult living kidney donors and recipients with prospectively registered perioperative data, including the donor and recipient profiles, were evaluated without any exclusions. Among donor surgeries, not more than three complications, requiring surgical (*n* = 1) or radiologic (*n* = 2) intervention without relation to moon phases were identified. As a consequence, no reliable conclusion could be drawn by the analysis of the live kidney donors in our cohort. Therefore the donors were not included in further analyses. The recipients’ age varied between 17 and 73 years with exception of three paediatric patients who were 7, 9, and 14 years old at the time of transplantation.

### Transplantation legislation

All transplantations were performed in accordance with the legislation of Eurotransplant and Germany. The medical eligibility of donors and recipients was evaluated by primary care physicians and nephrologists. Patients’ registration flowcharts were reviewed by an interdisciplinary review board of our institution and by an independent psychologic review board before transplantation. The study was approved by the local committee of ethics (grant no. 17–151).

### Surgical technique and data assessment

Surgical procedures were standardized as previously described [13, 14]. In the case of chronic graft failure, the graft was left in situ unless graft-related complications occurred. At our center, the prospective recording of a recipient’s data starts at the time of Eurotransplant waiting list registration. Data are updated every 6 months. Additional information about the clinical post-transplant course not included in the database was obtained in a 3-step process: information was extracted from the institution’s electronic patient files, checked, and then double checked by independent coworkers. All patient- and clinical data were recorded prospectively; moon phases and moon signs were assigned retrospectively to the date of KT. The following study provides consistent information over a minimum of 3-years of follow-up post transplantation, although all patients were registered for longer than 5 years (i.e. in the context of their first transplantation). Analyses were carried out in complete agreement with GCP guidelines for retrospective analyses [15] and, as far as applicable, according to the suggestions of the ISPOR Task Force on Retrospective Databases for the critical assessment of retrospective analyses [16].

### Timing of surgery, moon phases and zodiac signs

**Fig. 2:**
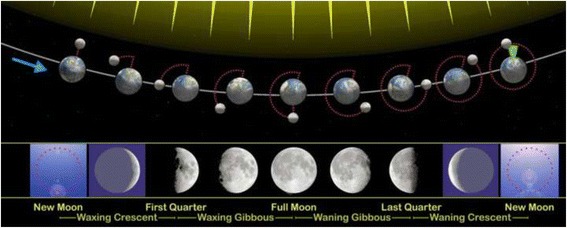
Moon phases as seen looking southwards from the northern hemisphere (upper part of the diagram not to scale, as the Earth-Moon distance is much bigger than shown here; © Orion 8/Wikimedia Commons/CC-BY-SA-3.0)

The majority of the transplant procedures had been planned irrespective of the moon cycle and moon signs. All surgeries were performed electively without exception. Therefore, all dates of LDKT were retrospectively assigned to the corresponding dates of the four lunar phases (Fig. 2). Lunar phases were defined as follows: the period of the new moon was considered to be ±1 day around the precise day of the new moon, so the entire new moon phase occupied 3 days. The full moon phase was defined similarly. The time interval between the new moon phase and the full moon phase was defined as the waxing moon phase and the time interval between the full moon phase and the new moon phase as the waning moon phase. Since a lunar month (synodic month) lasts about 29.53 days, the phases of waxing moon and of waning moon last approximately 12 days and therefore 4-times longer than the phases of full moon and new moon (3 days). Moon signs were defined as the signs of the Zodiac in which the moon was at the time of the operation. Lunar phases and moon signs for each date of transplantation were acquired from StarDate Online (online service of the University of Texas McDonald Observatory) [17]. According to medical astrology, the moon sign Libra is considered critical for renal and urogenital surgery (Fig. 1). Therefore, we additionally divided the patient cohort into two groups: those who underwent LDKT during the moon sign Libra, and those who did not.

### Immunosuppressive regimen

Graft recipients were treated with cyclosporine A (3 mg/kg) and prednisolone (250 mg). In addition, immunosuppression included azathioprine (2 mg/kg) or mycophenolate mofetil (MMF, 3 × 1,000 mg/d). Anti-thymocyte globuline (ATG-Fresenius®, 4 mg/kg) was administered as an induction regimen. Patients with re-transplantations were considered to be at a higher immunological risk and received tacrolimus (initial dose: 0.01 mg/kg) and 20 mg basiliximab plus ATG (1 mg/kg) as an induction regimen unless the preformed antibody ratio (PRA) was below 20%, according to the centers immunosuppressive protocol. Cytomegalovirus (CMV) prophylaxis was administered in all patients unless both the donor and the recipient were known to have a CMV-negative serologic status.

### Outcome measures definition

The following data were collected for each patient with regard to the date of transplantation: age, gender, total duration of dialysis, duration of the surgical procedure, postoperative morbidity and mortality, graft function (serum creatinine levels), rejection episodes, length of hospital stay, long-term complications, graft and patient survival rates, and causes of graft failure and/or patient death. Acute rejections were biopsy-proven and classified according to the BANFF ‘07 classification [18]. For statistical analysis, only rejections grade 3 and 4 (IA, B and IIA, B) were taken into account. Delayed graft function was defined as need for dialysis, or the absence of a spontaneous drop in serum creatinine within 7 days of transplantation after ruling out accelerated rejection, vascular complications, or urinary tract obstruction [19].

Postoperative morbidity was defined as any postoperative surgical or general complication following transplantation. Postoperative surgical complications such as vascular complications, ureteral complications, events of graft rupture and dislocation, development of lymphoceles, peritonitis, postoperative hemorrhage, events of wound dehiscence, as well as superficial and deep wound infection were recorded as described previously [20, 21]. In addition, the following types of general complications were registered: cardiac events, respiratory events, urinary tract infection, gastrointestinal bleeding and multi-organ failure.

### Statistical analysis

Continuous variables are presented as median (range), and categorical variables as total number (percentage). The Kruskal-Wallis- or the Mann–Whitney-U test were used for all comparisons among continuous variables. Categorical variables were compared by Pearson’s Chi-square statistics or Fisher’s exact test for small sample sizes. A *p*-value of 0.05 or less in a two-tailed test was considered statistically significant. Survival was analyzed using Kaplan-Meier estimates, and survival rates were analyzed using the log-rank test. All statistical analyses were performed using a SPSS Package (PASW version 17.0, SPSS Inc. U.S.A).

## Results

### Moon phases and moon signs

Thirty-three LDKT (11.9%) were performed during the new moon, 111 (39.9%) during the waning moon, 32 (11.5%) during the full moon, and 102 (36.7%) during the waxing moon. Therefore, all transplant procedures were distributed homogeneously among the main moon phases: new moon + waxing moon with 51.8% and full moon + waning moon with 48.2%. Seventeen of 273 transplantations (6.2%) were performed during the moon sign Libra.

### Demographics and medical history

Demographics and preoperative clinical data of all patients are shown in Table 1. Of the 278 patients, 192 (69.1%) were male and 86 (30.9%) were female. The median age and the median body mass index of all recipients were 42 years and 23.8 kg/m2, respectively. The median total duration of dialysis prior to LDKT was 1.96 years, and 30 patients (10.8%) underwent peritoneal dialysis before. Main causes of terminal kidney failure prior to transplantation were chronic glomerulonephritis (*n* = 88, 31.7%), IgA nephritis (*n* = 24, 8.6%), polycystic kidney disease (*n* = 19, 6.8%), and diabetic nephropathy (*n* = 13, 4.7%). Thirty-three patients (11.9%) had more than one kidney transplantation prior to LDKT. All patients were clinically followed up for a median duration of 8.2 years after LDKT. No differences were seen between all groups of lunar phases and the group of the moon sign libra, with respect to patient’s demographics, underlying kidney disease, medical- and transplant history (Table 1).

**Table 1:** Demographics and medical history of LDKT recipients

		Patient groups according to moon phases/moon sign Libra					*P*-value*
	n (%) ^a^	New moon	Waxing moon	Full moon	Waning moon	Moon sign Libra	
Demographics							
Number of patients	278 (100.0)	33 (100.0)	111 (100.0)	32 (100.0)	102 (100.0)	17 (100.0)	
Age (years) ^b,c^	42.0 [7.4 - 74.0]	45.3 [7.6 - 68.8]	40.0 [17.8 - 73.9]	42.1 [7.4 - 69.0]	45.6 [9.3 - 70.3]	35.9 [23.1 - 61.6]	0.213
Sex (m/f)	192 (69.0)/86 (31.0)	28 (84.8)/5 (15.2)	74 (66.7)/37 (33.3)	23 (71.9)/9 (28.1)	67 (65.7)/35 (34.3)	12 (70.6)/5 (29.4)	0.186
BMI (kg/m^2^) ^c^	23.8 [16.4 - 34.8]	24.5 [16.7 - 30.9]	23.9 [16.4 - 33.8]	22.9 [17.2 - 30.9]	23.8 [16.9 - 34.8]	23.9 [17.4 - 28.0]	0.784
Medical history							
Total duration of dialysis (years) ^b,c^	1.96 [0.04 - 10.64]	1.75 [0.04 - 7.32]	2.02 [0.2 - 10.6]	1.8 [0.2 - 7.7]	2.3 [0.04 - 10.5]	2.4 [0.7 - 10.6]	0.606
Peritoneal dialysis	30 (10.8)	0 (0)	14 (12.6)	3 (9.4)	13 (12.7)	3 (17.6)	0.176
Cause of kidney failure ^d^							
Chronic glomerulonephritis	88 (31.7)	8 (24.2)	32 (28.8)	11 (34.4)	37 (36.3)	6 (35.3)	0.500
IgA nephritis	24 (8.6)	4 (12.1)	10 (9.0)	2 (6.3)	8 (7.8)	0 (0)	0.840
Polycystic kidney disease	19 (6.8)	2 (6.1)	8 (7.2)	2 (6.3)	7 (6.9)	3 (17.6)	0.995
Diabetic nephropathy	13 (4.7)	2 (6.1)	8 (7.2)	1 (3.1)	2 (2.0)	2 (11.8)	0.308
Transplantation history							
one or more KT prior to LDKT	33 (11.9)	3 (9.09)	14 (12.6)	3 (9.4)	13 (12.7)	2 (11.8)	0.915
Follow up							
Follow-up after LDKT (years) ^c^	8.2 [0.13 - 15.2]	8.1 [1.9 - 12.6]	7.9 [0.13 - 14.6]	10.4 [0.8 - 15.2]	8.3 [0.6 - 14.2]	6.0 [1.9 - 12.3]	

*KT* kidney transplantation

*LDKT* living donor kidney transplantation

^*^ *p*-values are calculated for moon phases only

^a^values are number (percentage) unless indicated otherwise

^b^data are presented with respect to the date of LDKT

^c^values are median [range]

^d^most common causes

### Postoperative course

**Fig. 3:**
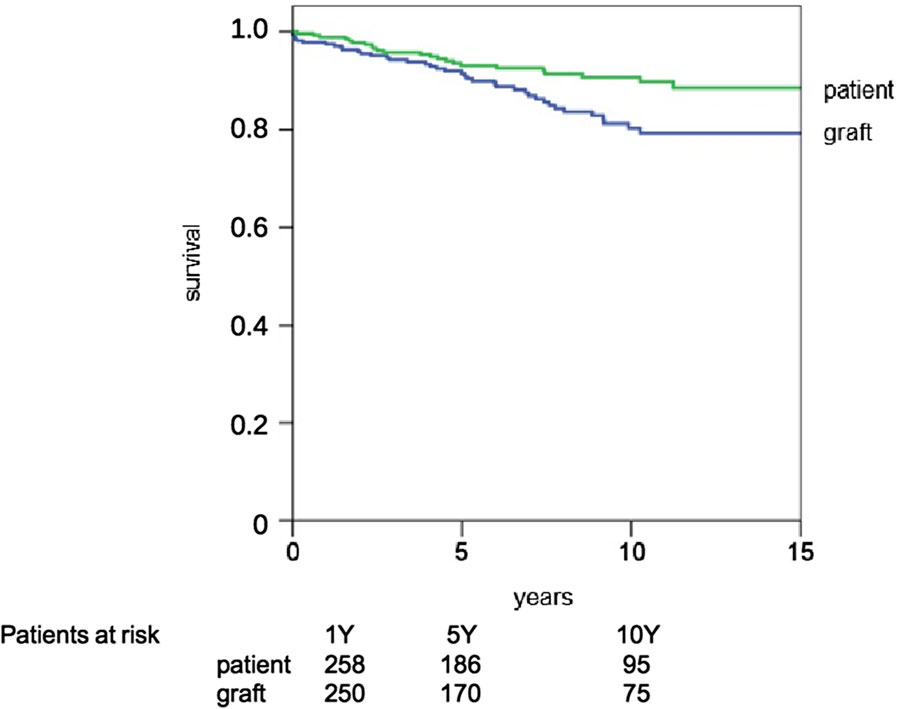
Patient- and graft survival after living donor kidney transplantation (LDKT). Patient- and graft-survival rates at 1, 5, and 10 years after transplantation were 98.9, 92, and 88.7% and 97.4, 91.6, and 80.6%, respectively

**Fig. 4:**
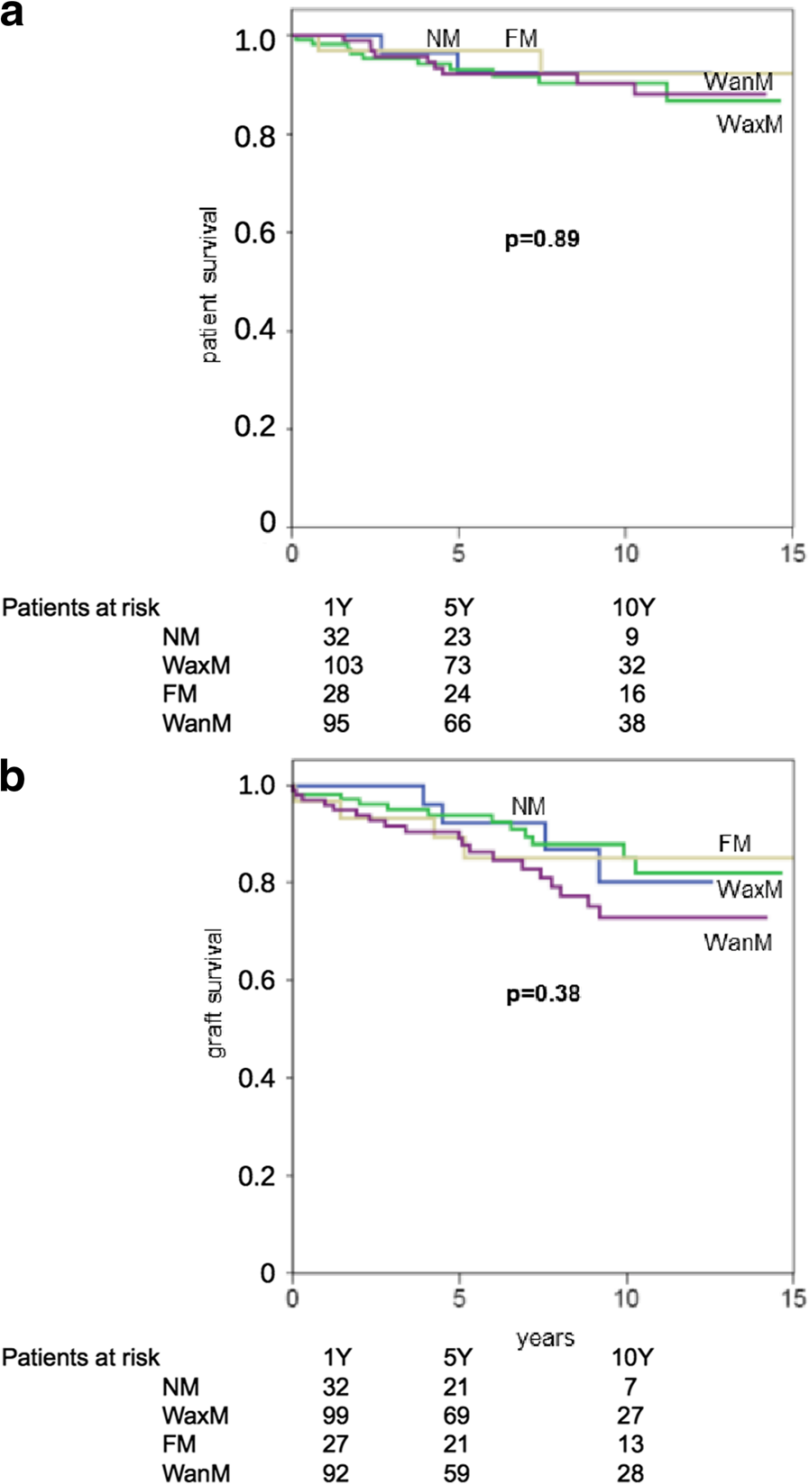
**a** Patient- and (**b**) graft survival after living donor kidney transplantation (LDKT) in recipients, grouped according to the four main moon phases: *new moon* (NM); *waxing moon* (WaxM); *full moon* (FM) and *waning moon* (WanM). The *full moon phase*, which, according to medical astrology, should be avoided for operations, did not show a worse course than other moon phases

**Fig. 5:**
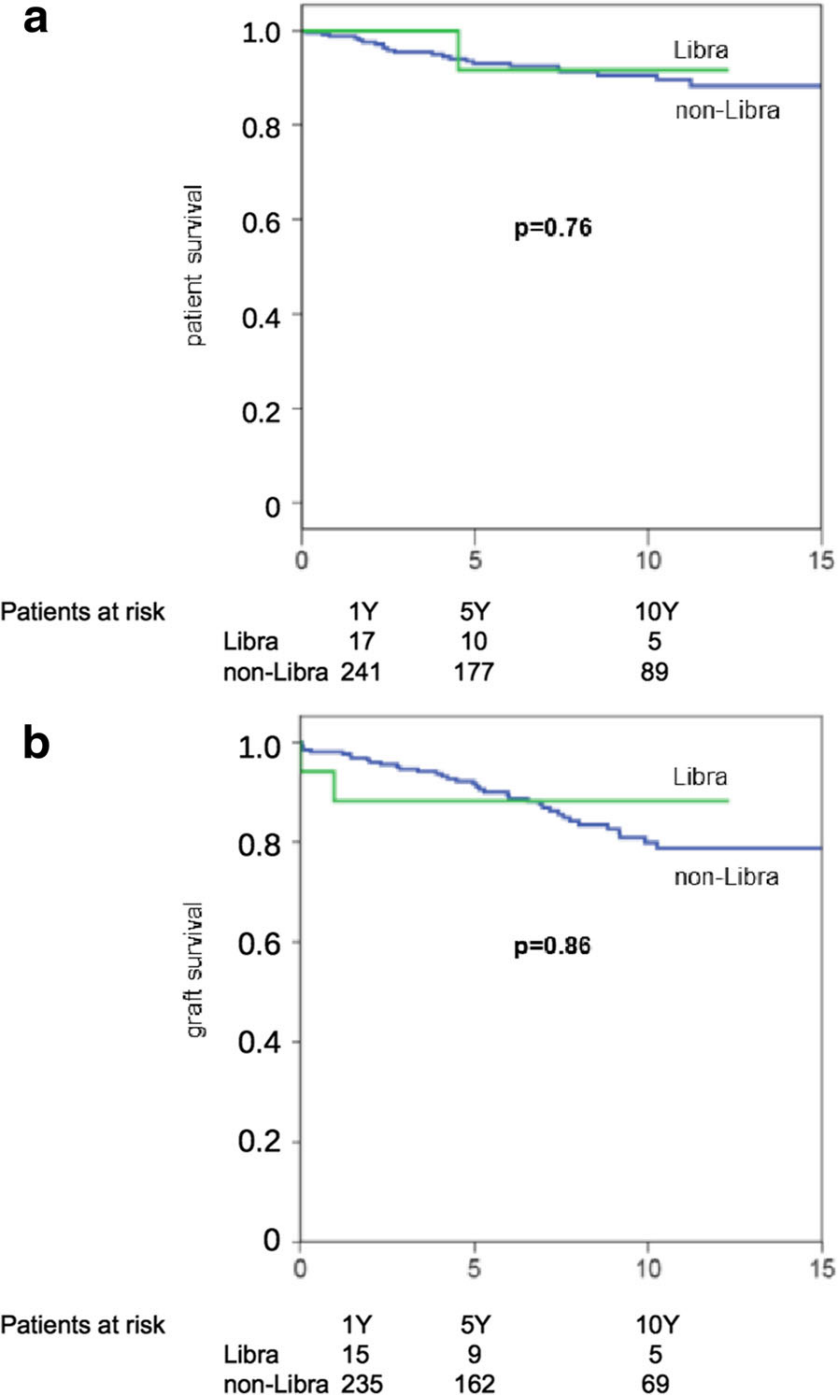
**a** Patient- and (**b**) graft survival after living donor kidney transplantation (LDKT) in recipients, grouped according to the moon sign *Libra*, which, according to medical astrology, is believed to interfere with surgical procedures of the kidneys and the urinary tract

Operative data and the clinical course of patients are shown in Table 2. No significant differences between all groups were found regarding duration of the procedure, postoperative length of stay, postoperative morbidity, rejection episodes, graft loss and 90-day-mortality. Overall, 59 patients (21.2%) developed postoperative surgical complications, and 26 patients (9.4%) demonstrated delayed graft function without significant differences across the study groups; early graft loss (<30 days after transplantation) occurred in four patients (1.4%). With regard to the date of LDKT, patient and graft survival rates at 1, 5, and 10 years were 98.9, 92, and 88.7%, and 97.4, 91.6, and 80.6%, respectively (Fig. 3). The timing of surgery with respect to the moon phases and the moon sign Libra showed no significant impact on long-term patient and graft survival (Figs. 4 and 5). Taken together, we cannot demonstrate any influence of moon phases and the moon sign Libra on the peri- and postoperative course of LDKT patients. In contrast to graft recipients, live donors revealed postoperative complications which required surgical or radiological revision in only 1.1% without relation to moon phases or Libra sign.

**Table 2:** Operative data and postoperative complications of LDKT recipients

		Patient groups according to moon phases/moon sign Libra					*P*-value^*^
	n (%) ^a^	New moon	Waxing moon	Full moon	Waning moon	Moon sign Libra	
Number of patients	278 (100.0)	33 (100.0)	111 (100.0)	32 (100.0)	102 (100.0)	17 (100.0)	
Duration of operation (min.) ^b^	160 [65–345]	155 [120–345]	160 [90–280]	155 [65–295]	160 [99–335]	160 [120–265]	0.532
Graft placement ^c^:							
R-L	133 (47.8)	16 (48.4)	58 (52.3)	17 (53.1)	42 (41.2)	11 (64.7)	0.358
L-R	91 (32.7)	13 (39.4)	32 (28.8)	9 (28.1)	37 (36.3)	4 (23.5)	0.530
L-L	23 (8.3)	3 (9.1)	8 (7.2)	2 (6.3)	10 (9.8)	1 (5.9)	0.883
R-R	31 (11.2)	2 (6.1)	12 (10.8)	4 (12.5)	13 (12.7)	1 (5.9)	0.756
Hospital length of stay (days) ^b^	18 [1–65]	20 [7–43]	17.5 [5–65]	16.5 [7–58]	18 [1–53]	21 [6–32]	0.851
General complications during inhospital stay ^d,e^	12 (4.3)	1 (3.0)	4 (3.6)	2 (6.3)	5 (4.9)	0 (0)	0.887
Surgical complications during inhospital stay ^f^	59 (21.2)	10 (30.3)	23 (20.7)	6 (18.8)	20 (19.6)	5 (29.4)	0.589
Relaparotomy	49 (17.6)	8 (16.2)	18 (16.2)	6 (18.8)	17 (16.7)	4 (23.5)	0.744
Rejection episodes ^g^	56 (20.1)	8 (24.2)	24 (21.6)	8 (25.0)	16 (15.7)	1 (5.9)	0.527
Delayed graft function ^h^	26 (9.4)	1 (3.0)	11 (9.9)	2 (6.3)	12 (11.8)	0 (0)	0.447
Long-term surgical complications ^i^ (>90 days)	25 (9.0)	2 (6.1)	11 (9.9)	3 (9.4)	9 (8.8)	1 (5.9)	0.925
Mortality:							
(<30 days)	0 (0)	0 (0)	0 (0)	0 (0)	0 (0)	0 (0)	
(<90 days)	1 (0.4)	0 (0)	1 (0.9)	0 (0)	0 (0)	0 (0)	
Graft loss:							
(<30 days)	4 (1.4)	0 (0)	2 (1.8)	1 (3.1)	1 (1.0)	1 (5.9)	
(<90 days)	5 (1.8)	0 (0)	2 (1.8)	1 (3.1)	1 (1.0)	1 (5.9)	

*KT* kidney transplantation, *LDKT* living donor kidney transplantation

^*^ *p*-values are calculated for moon phases only.

^a^values are number (percentage) unless indicated otherwise

^b^values are median [range]

^c^L = left, R = right, the first letter indicates the kidney, the second letter indicates the placement side

^d^including cardiac events, respiratory events, multiorgan failure and gastrointestinal bleeding

^e^some patients had more than one complication

^f^including vascular complications, ureteral complications, events of graft rupture and dislocation, developmant of lymphocele, peritonitis, postoperative hemorrhage, events of fascial dehiscence, superficial and deep wound infection

^g^all rejection episodes were biopsy-proven

^h^need for dialysis in the first 7 days after transplant and/or the presence of biopsy-proven acute tubular necrosis (ATN)

^i^including vascular, urologic complications, fascial dehiscence, hernia

## Discussion

The present single center cohort study could reveal that timing of transplant surgery according to moon phases or the moon sign Libra generally had no measurable impact on short- and long-term outcome after living donor kidney transplantation.

Even in modern ‘informed’ societies, superstition still plays a significant role. Only a small proportion of people approach life in an entirely rational way [22]. Since superstition is in general often tied to unpredictable events beyond personal control, people may rely on astrology to predict the future. Even today, the concept of lunar phases influencing different aspects of daily life is very popular. The moon phase hypothesis, almost completely abandoned by the medical field since 1700 [23], had a renaissance in the second half of the 20th century after a report by Andrews in 1960 who observed an increased incidence of bleeding complications after tonsillectomies carried out during the full moon [8]. Studies in terms of an influence of the moon phases on human life thereafter were inconclusive [9, 10, 24–34], or failed to confirm it [35–38]. Nevertheless, several studies demonstrated significant differences in social life, for example in alcohol consumption, nutritional intake, birth rates, and the frequency of incidents such as crime, self-poisoning, suicide and traffic accidents, during the four moon phases [25, 31–34]. Moreover, general practice consultation rates and hospital admissions for anxiety, depression, acute diarrhea, gastrointestinal bleeding, atrial fibrillation, myocardial infarction and cardiac arrest have been found to vary throughout the lunar cycle [9, 10, 24, 26–30]. These reconfirmed a presumed dependence of social life and humans physical condition on a superordinate authority such as moon phases and moon signs. Accidents, violence, births rates and acute illnesses, however, can hardly be influenced or timely scheduled by humans. In the same sense, postoperative complications following transplantation in general occur by hazard. Therefore these results remain observantly. With regard to elective treatment, however, the question arises of how the physical condition of the doctor on one hand, and how the patients healing course on the other hand might be influenced by different phases of the moon. Furthermore, a specific timing of the treatment might improve the patient’s outcome. About 10.5% of the German population believes that particular moon phases influence disease onset, clinical course, and recovery. This number is even higher in the South of Germany where our transplant center is located. Here, up to 17.9% believe in the moon phase hypothesis [11]. In this context no clear scientific opinion on the influence of lunar phases on surgical outcome exists, and only few data is available to physicians and surgeons with which to relieve the patients’ concerns about wrong timing of surgery. Some studies have analyzed surgical outcome as a function of moon phases. Most of these studies could demonstrate no relationship between the lunar cycle and postoperative outcome after elective ambulantory surgery, surgery performed under general anesthesia, or more specific after breast cancer-, lung cancer-, bladder cancer- or ENT surgery [11, 36–39].

The reason for the present study was due to an increasing patient demand for moon-phase-adjusted transplant appointments in our own department. Especially in transplantation medicine, the mental and psychological stress of all participants, especially donors and recipients is very high. Factors, such as the live organ of a closely related party, the threat of a life-threatening illness, the vague hope of cure but also the knowledge about the limited life-span of the new organ,put the patients under enormous pressure. All thinkable efforts are in general made in order to increase the likelihood of a perfect long term organ survival. Not surprisingly, our patient managers have to consult lunar calendars during the planning of LDKT appointments with increasing frequency. However, it is not always possible to accommodate the patients’ wish dates.

The present study has some limitations. First, it is a retrospective analysis of a single transplant center and the number of patients is limited. In addition, due to the retrospective design of our study, no randomization of the patients was realized.. However, a randomization of the patients would systematically ignore the patients preferences. Realization of such a randomized trial would therefore be challenging. As a consequence, we do not know in how many cases the transplant was scheduled according to the lunar phases on the explicit request of the patient. It would be interesting to know whether a strong desire of the patient and a corresponding fulfillment has any influence on the short- and long-term success of the surgery. It cannot be excluded that the patient’s fear of wrong timing of the transplantation might have a significant negative influence on postoperative recovery and outcome, irrespective of any influence of the moon.

However, the results of the present study revealed that the moon phases and the position of the moon in the moon sign Libra generally had no measurable impact on short- and long-term outcome after LDKT. These results are suitable to ease recipients, donors and relatives, if a transplantation procedure according to the moon cycle is not possible.

## Conclusion

Our data indicate that there is no connection between moon phases, moon signs and results after living donor kidney transplantation (LDTK). On the other hand, physicians and surgeons should consider their patients’ preferences, wishes, and beliefs first. Therefore, patients who strongly confide in the impact of the moon phases on the outcome of surgery should be taken seriously and the correct timing of transplantation should be facilitated as long as it does not interfere with evidence-based treatment and center-specific regimens.

## Abbreviations

FM: Full Moon
KT: Kidney transplantation
LDKT: Living Donor Kidney Transplantation
NM: New Moon
WanM: Waning Moon
WaxM: Waxing Moon
