# Correction to: Moon phases and Moon signs do not influence morbidity, mortality and long-term survival, after living donor kidney transplantation

**DOI:** 10.1186/s12906-017-1981-z

**Published:** 2017-10-04

**Authors:** A. Kleespies, M. Mikhailov, P. N. Khalil, S. Pratschke, A. Khandoga, M. Stangl, W. D. Illner, M. K. Angele, K. W. Jauch, M. Guba, J. Werner, M. Rentsch

**Affiliations:** Department of General-, Visceral-, Vascular- and Transplant Surgery, University of Munich, Marchioninistrasse 15, 81377 Munich, Germany

## Erratum

The authors of this article [1] have stated that the image in Fig. 1 in the main article is incorrect and would like readers to refer to the image in this correction instead.

**Fig. 1:**
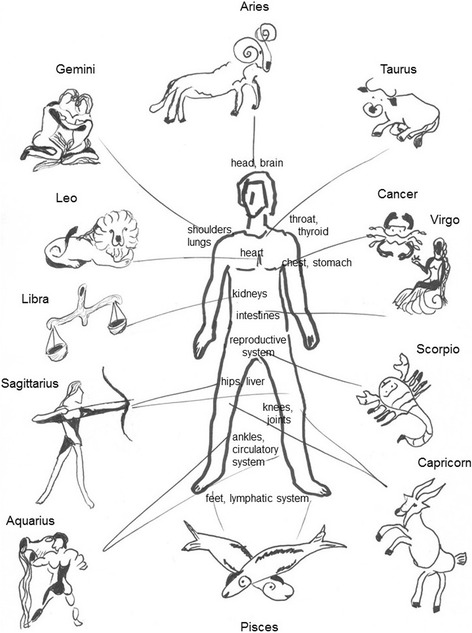
Anatomical-astrological human (drawing by © Alla Mikhailova). According to medical astrology, each organ (or organ system) is associated with a certain moon sign. Note, that the kidneys are assigned to the sign of *Libra*
